# Use of a Smart Watch for Early Detection of Paroxysmal Atrial Fibrillation: Validation Study

**DOI:** 10.2196/14857

**Published:** 2020-01-22

**Authors:** Tomohiko Inui, Hiroki Kohno, Yohei Kawasaki, Kaoru Matsuura, Hideki Ueda, Yusaku Tamura, Michiko Watanabe, Yuichi Inage, Yasunori Yakita, Yutaka Wakabayashi, Goro Matsumiya

**Affiliations:** 1 Department of Cardiovascular Surgery University of Chiba Chiba Japan; 2 Clinical Research Center University of Chiba Chiba Japan

**Keywords:** Apple Watch, Fitbit Charge HR, paroxysmal atrial fibrillation, photoplethysmography, mobile health, heart rate, validation, wrist-banded devices

## Abstract

**Background:**

Wearable devices with photoplethysmography (PPG) technology can be useful for detecting paroxysmal atrial fibrillation (AF), which often goes uncaptured despite being a leading cause of stroke.

**Objective:**

This study is the first part of a 2-phase study that aimed at developing a method for immediate detection of paroxysmal AF using PPG-integrated wearable devices. In this study, the diagnostic performance of 2 major smart watches, Apple Watch Series 3 and Fitbit (FBT) Charge HR Wireless Activity Wristband, each equipped with a PPG sensor, was compared, and the pulse rate data outputted from those devices were analyzed for precision and accuracy in reference to the heart rate data from electrocardiography (ECG) during AF.

**Methods:**

A total of 40 subjects from patients who underwent cardiac surgery at a single center between September 2017 and March 2018 were monitored for postoperative AF using telemetric ECG and PPG devices. AF was diagnosed using a 12-lead ECG by qualified physicians. Each subject was given a pair of smart watches, Apple Watch and FBT, for simultaneous pulse rate monitoring. The heart rate of all subjects was also recorded on the telemetry system. Time series pulse rate trends and heart rate trends were created and analyzed for trend pattern similarities. Those trend data were then used to determine the accuracy of PPG-based pulse rate measurements in reference to ECG-based heart rate measurements during AF.

**Results:**

Of the 20 AF events in group FBT, 6 (30%) showed a moderate or higher correlation (cross-correlation function>0.40) between pulse rate trend patterns and heart rate trend patterns. Of the 16 AF events in group Apple Watch (workout [W] mode), 12 (75%) showed a moderate or higher correlation between the 2 trend patterns. Linear regression analyses also showed a significant correlation between the pulse rates and the heart rates during AF in the subjects with Apple Watch. This correlation was not observed with FBT. The regression formula for Apple Watch W mode and FBT was X=14.203 + 0.841Y and X=58.225 + 0.228Y, respectively (where X denotes the mean of all average pulse rates during AF and Y denotes the mean of all corresponding average heart rates during AF), and the coefficient of determination (*R*^2^) was 0.685 and 0.057, respectively (*P*<.001 and .29, respectively).

**Conclusions:**

In this validation study, the detection precision of AF and measurement accuracy during AF were both better with Apple Watch W mode than with FBT.

## Introduction

### Background

Atrial fibrillation (AF) is the most common sustained arrhythmia that afflicts approximately 34 million people worldwide. AF is a well-known risk factor for stroke, with almost one-third of all strokes being attributed to this arrhythmia [1-5]. Moreover, nearly one-third of patients with paroxysmal AF are asymptomatic [1,4-6], thus obscuring the diagnosis of this arrhythmia; in fact, up to 50% of patients with stroke caused by AF are diagnosed with AF after the onset of a stroke event [7-9].

With the advent of mobile devices and wearable sensors, it has become possible to continuously monitor health in daily life. Over 450 million wearable devices have been sold, and the current sales growth rate of these devices is approximately 20% per year [10,11]. Among these devices, the smart watch has been gaining attention for its potential usefulness as a wristband-type continuous pulse measurement terminal. This device carries a photoplethysmograph, a photodetector that uses infrared light-emitting diode optical sensors to monitor blood volume changes of the microvasculature [12].

Photoplethysmography (PPG) allows pulse rate to be passively and continuously computed on the smart watch. Each pulse signal captured by PPG can be interpreted as an R wave on the electrocardiogram [12]. If the R wave in AF can be detected with high precision using PPG, it would be possible to diagnose AF based on the pulse rate [13,14]. Therefore, an algorithm to detect AF using PPG would be an attractive alternative to existing electrocardiography (ECG)–based monitoring, which has limitations, particularly in patients with asymptomatic paroxysmal AF [15-19].

The clinical applicability of PPG has been addressed in many studies, with most studies demonstrating high accuracy in PPG-based pulse measurement among healthy subjects who have no arrhythmia [20-22]. However, ambiguities exist regarding the accuracy of the pulse measurements in patients with an arrhythmia, particularly AF. In addition, the usefulness of PPG as a diagnostic tool for detecting AF has remained inconclusive as most reports were based on a short observation period and under resting conditions in patients suffering from persistent AF [23]. Importantly, those studies have not taken into account motion artifacts and other noises that may occur regularly in daily life [24]. Characteristic signals or patterns suggesting the onset and offset of AF have also not been determined [23-26].

### Objectives

The primary purpose of this study was to develop a method for immediate detection of paroxysmal AF using PPG technology and to determine whether PPG-based diagnosis of paroxysmal AF is feasible in clinical practice. To achieve this, we divided the study into 2 parts: (1) validation of precision and accuracy of data acquired from PPG and (2) development of an algorithm for on-the-spot detection and diagnosis of paroxysmal AF. This paper, which represents the first part of this study, compared the diagnostic performance of 2 major PPG-integrated smart watches, Apple Watch Series 3 (Apple Inc) and Fitbit (FBT) Charge HR Wireless Activity Wristband (Fitbit Inc), and assessed whether pulse rate values and variations obtained from the PPG devices can help detect paroxysmal AF. Given the high incidence of paroxysmal AF in patients early after cardiac surgery [27-29], those patients were chosen as our study subjects.

## Methods

### Study Protocol

This study was approved by the Clinical Research Ethics Committee of Chiba University Hospital (protocol number UMIN000028403; approved July 27, 2017) in accordance with all applicable regulations. All study subjects provided written informed consent that allowed data monitoring, which was performed by the Chiba University Hospital Clinical Trials Data Center, and data registration and management, which were undertaken by the University of Tokyo. An independent data monitoring committee was also established within the Clinical Trials Division, Chiba University.

From September 2017 to March 2018, 40 subjects from patients scheduled for cardiac surgery at a single center were recruited for part 1 of this study. The exclusion criteria for this study were a history of permanent pacemaker implantation, skin disorder at the wristband attachment site, rubber allergy, and postoperative pacemaker requirement.

After obtaining written informed consent, the 40 subjects were given a pair of smart watches, Apple Watch and FBT, which were worn side by side on one forearm. A fully charged extra pair of smart watches was made available at all times in case an exchange was needed and to prevent data loss. The exchange was always carried out by a doctor to ensure data continuity. The smart watches were given to the subjects when the subjects were freed from intensive care (usually on the next day after surgery). The watches were worn continuously until discharge or for 2 weeks, unless the study was aborted for clinical or personal reasons.

Apple Watch offers 2 functional modes with different algorithm settings, the standby (S) mode and the workout (W) mode. Each mode also differs in the algorithm for pulse rate measurements (as described below), and the subjects were monitored using 1 of the 2 modes depending on when the device was given. Subjects who started wearing the device before November 2017 were monitored with the S mode until the end of their observation period. From November 2017, the W mode was used instead. The following groups were thus formed: 40 subjects with group FBT, 18 subjects with Apple Watch S mode (group AWS), and 22 subjects with Apple Watch W mode (group AWW).

Central ECG monitoring using a telemetry system (DynaBase CVW-7000, Fukuda Denshi) was continued in all patients until discharge. If AF was suspected, a 12-lead ECG was performed for confirmation [30]. AF was diagnosed based on the guided diagnostic criteria by a qualified physician. When AF was confirmed, its onset and offset were recorded by reviewing the telemetry data. These procedures were repeated whenever AF was suspected on the central monitor. Any drug therapy that was initiated after AF occurrence was also recorded.

### Heart Rate and Pulse Rate Measurements

Heart rate data were obtained from the telemetric electrocardiograph, which calculates heart rate every second based on the immediately preceding RR interval.

Pulse rate data were obtained from the PPG-integrated smart watches, although the algorithm for pulse measurements differs slightly between devices. FBT calculates the pulse rate by taking the average of the pulse signals captured between 2 and 5 seconds. Apple Watch has 2 functional modes with different algorithm settings: on S mode, the pulse rate (average of pulse signals) is computed at roughly every 6 min, and on W mode, the rate is calculated every 5 to 6 seconds. In addition, Apple Watch has an automatic optimization function that increases the luminance of the light-emitting diode and sampling rate to compensate for low signal levels (eg, low perfusion states and dark skin tones); therefore, the time interval (Δt) between each pulse rate calculation on Apple Watch may fluctuate depending on conditions [31].

Both heart rate and pulse rate data were outputted as a comma-separated values file for subsequent analyses.

### Cross-Correlation Analysis

**Figure 1:**
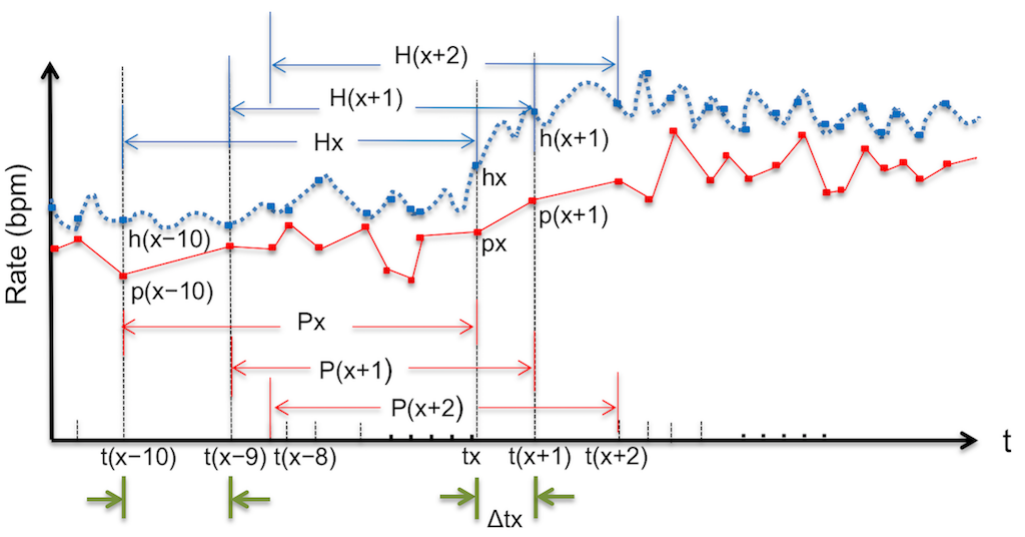
Schematic diagram of time series curves with matching measurement intervals (Step1). The figure shows the pulse rates (px) calculated on the smart watches and the corresponding heart rates (hx) on the electrocardiographic monitor at each time point (tx) when the pulse rate was calculated. The time interval (Δt) shown on this graph is dependent on the pulse rate measurements. The average of 10 consecutive pulse rates and 10 corresponding heart rates is Px and Hx, respectively. bpm: beats per minute.

**Figure 2:**
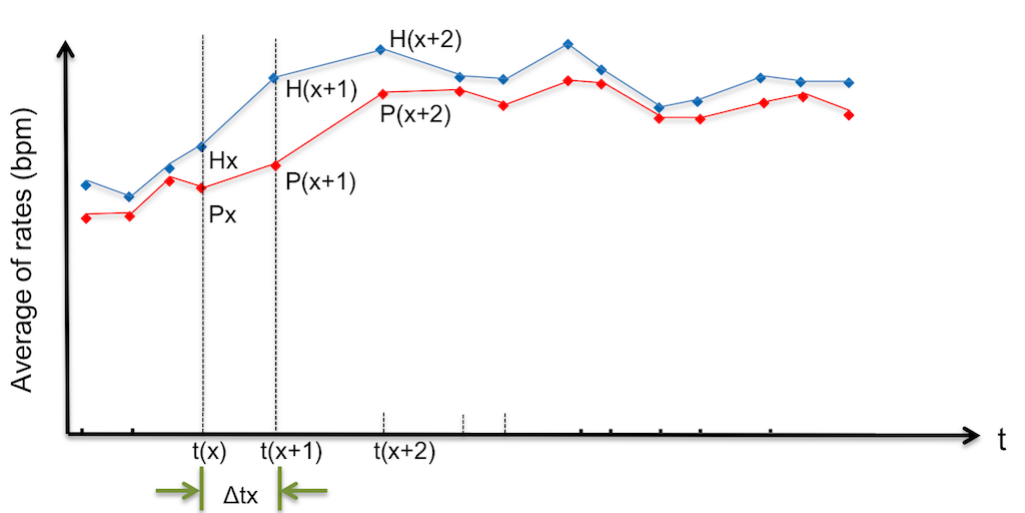
Schematic diagram of time series curves with matching measurement intervals (Step2). The figure shows the averages of the pulse rates (Px) and the averages of the corresponding heart rates (Hx). These trend curves were used for subsequent analyses. The red dot represents pulse rate, and the blue dot represents heart rate (bolded blue dot represents corresponding heart rate). bpm: beats per minute; t: time.

To validate the data obtained from the PPG devices for the detection of AF, the pulse rate data from the PPG devices were compared with the heart rate data from telemetric ECG. Given the variability in the time interval (Δt) for pulse rate calculation as opposed to the time interval, which is constant at 1 second, for heart rate calculation, we created a time series graph (Figure 1) showing the pulse rate {px, p(x+1), p(x+2),...} and the corresponding heart rate {hx, h(x+1), h(x+2),...} at each time point {tx, t(x+1), t(x+2),...} when the pulse rate was calculated. To adjust for the differences in the time intervals, we took the average of all pulse rates calculated within a time frame, for example, {t(x-10) to t(x)} and compared that with the average of the corresponding heart rates calculated within the same time frame. The comparison was repeated by shifting the time frame forward by 1 time point {t(x-9) to t(x+1), t(x-8) to t(x+2),...}. Thus, a similar time series graph (Figure 2) that compared the averages of the pulse rates {Px, P(x+1), P(x+2),...} and the averages of the corresponding heart rates {Hx, H(x+1), H(x+2),...} can be drawn. Those averages were used to analyze for similarities in various trend patterns in the trend curves of the heart rate and the pulse rate by determining their cross-correlation functions (CCFs), which range between −1 and 1. In general, the closer the CCF value is to 1, the more similar the patterns are.

In this CCF analysis, the time frame to determine the averages of the calculated rates was set to contain 10 consecutive pulse rate measurements. This time frame corresponded to approximately 1 min of recording time. Datasets (1 se*t* =10 pulse/heart rate data) that largely deviated from this 1-min time frame were excluded from this analysis. After creating the time series trend curves from the averaged rates, a CCF analysis was performed as follows: (1) apply single regression analysis to each time series data, (2) calculate the residuals at each time point, and (3) calculate the correlation coefficient by using the residuals of each time series data [32,33].

### Simple Linear Regression Analysis

To evaluate the accuracy of the pulse rates based on PPG measurement in reference to the heart rates based on ECG during AF, a simple linear regression analysis was performed using the same datasets created for the CCF analysis. The mean and the standard deviation of all average pulse rates {Px, P(x+1), P(x+2),...} during AF were compared with the mean and the standard deviation of the corresponding average heart rates {Hx, H(x+1), H(x+2),...}.

### Other Statistical Analyses

Summary statistics were constructed using frequencies and proportions for categorical data and mean (standard deviation) for continuous data. Comparisons between groups were carried out using Student *t* test, analysis of variance, or nonparametric tests for continuous data and Pearson chi-square test for categorical data. *P* values less than .05 were considered statistically significant.

All statistical analyses were performed using SAS version 9.4 for Windows (SAS Institute Inc).

## Results

### Patient Demographics

The demographics of the 40 study subjects are shown in Table 1.

**Table 1:** Characteristics of patients in each group (note that statistical comparison on this table is made only between Apple Watch standby mode and Apple Watch workout mode because of overlaps).

Demographics	Apple Watch standby mode (n=18)	Apple Watch workout mode (n=22)	Fitbit Charge HR (n=40)
Age (years), mean (SD)	71.0 (11.9)	70.7 (10.6)	70.9 (11.1)
Male, n (%)	11 (61)	16(72)	27 (68)
Left ventricular ejection fraction, mean (SD)	57.7 (13.2)	61.1 (10.2)	59.5 (11.6)
Off-pump coronary artery bypass grafting, n (%)	2 (11)	5 (23)	7 (18)
Valve surgery, n (%)^a^	14 (78)	13 (59)	27 (68)
Other surgery, n (%)^b^	2 (11)	4 (18)	6 (15)
Postoperative stay, days	24 (17.5)	17.5 (8.3)	20.7 (13.6)
Monitoring period, days	12.4 (2.1)	10.3 (2.1)	11.3 (2.7)
Use of antiarrhythmic drugs before event, n (%)^c^	16 (89)	22 (100)	38 (95)
Use of antiarrhythmic drugs after event, n (%)^d^	18 (100)	22 (100)	40 (100)

^a^Included multiple surgery.

^b^Included 2 thoracic surgeries, 1 atrial septal defect closure, and 3 on-pump beating coronary artery bypass graftings.

^c^Types of antiarrhythmic drugs included pilsicainide, amiodarone, verapamil, and beta-blockers.

^d^Types of antiarrhythmic drugs included pilsicainide, amiodarone, verapamil, and beta-blockers.

### Heart Rate and Pulse Rate Measurements

The number of times pulse rate was calculated on the PPG devices was 23,665, 1,758,226, and 4,791,577 in groups AWS, AWW, and FBT, respectively. The time interval (Δt) between each pulse rate calculation was, in seconds, 393.6 (525.7), 6.2 (6.1), and 3.5 (2.6) in groups AWS, AWW, and FBT, respectively. In particular, Δt for pulse rate varied from 1 second to 39 min in group AWS with no noticeable increase in the sampling rate during AF.

AF occurred in 24 out of 40 (60%) subjects. We detected 33 AF events, which included 5 in group AWS, 28 in group AWW, and 33 in group FBT, all confirmed by a 12-lead ECG as per guidelines. For validation purposes, very brief episodes of AF and AF events with unclear onset or offset were excluded. AF events that contained device-related noises and interruptions and those with wide Δt causing deviation from the CCF analysis criteria were also excluded. After the exclusion process, 23 AF events were considered fit for this validation study.

### Validation of Precision of Detecting Atrial Fibrillation: Cross-Correlation Analysis

Table 2 shows the results of the CCF analysis. The table lists the 23 AF events that can be used for the analysis. As shown, there were 20 and 16 events in groups FBT and AWW, respectively, and none in group AWS that met the analysis criteria.

Of the 20 AF events in group FBT, 9 showed a very weak or a negative correlation between pulse rate trend patterns and heart rate trend patterns. Of the 16 AF events in group AWW, 2 showed a very weak or a negative correlation between the 2 trend patterns. A comparison of the 2 groups by AF events (numbers 8-11, 14, 15, and 17-23 in Table 2) showed a stronger correlation with group AWW. Regarding group AWS, all 5 events that were confirmed positive for AF were excluded from the analysis because of the very low number of pulse rate measurement per given time frame.

**Figure 3:**
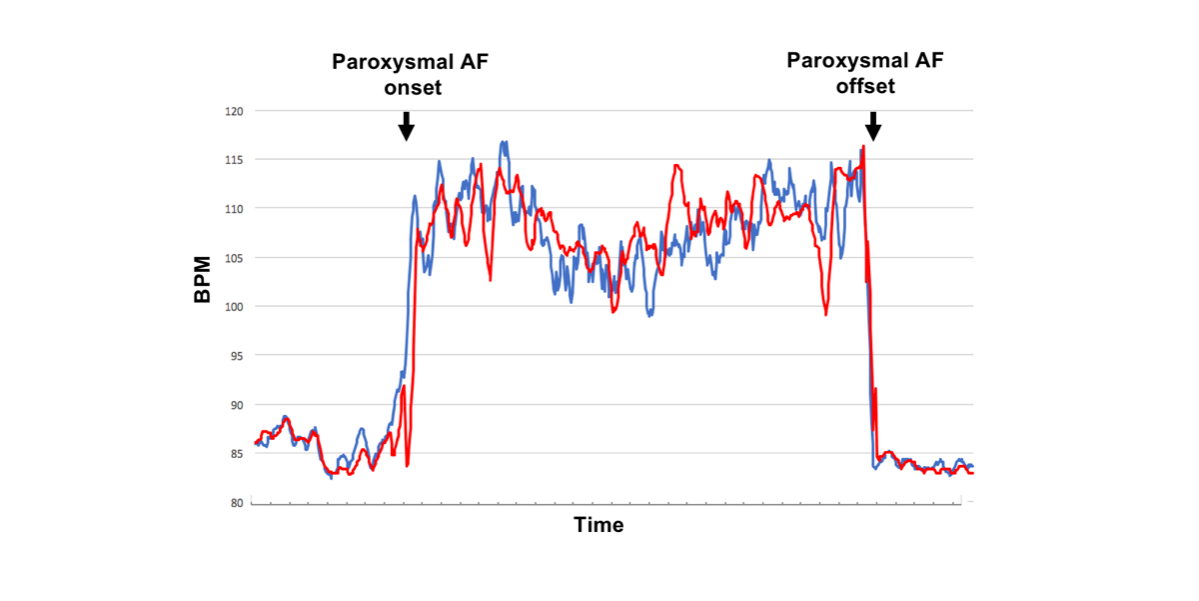
Time series trend curves during atrial fibrillation (event number 18). The figure compares the trend curve of the Apple Watch W mode pulse rate (red curve) with that of the electrocardiography-based heart rate (blue curve). The 2 trend curves follow a similar pattern and therefore appear almost identical (cross-correlation function 0.83; *P*&lt;.001). AF: atrial fibrillation; bpm: beats per minute.

**Figure 4:**
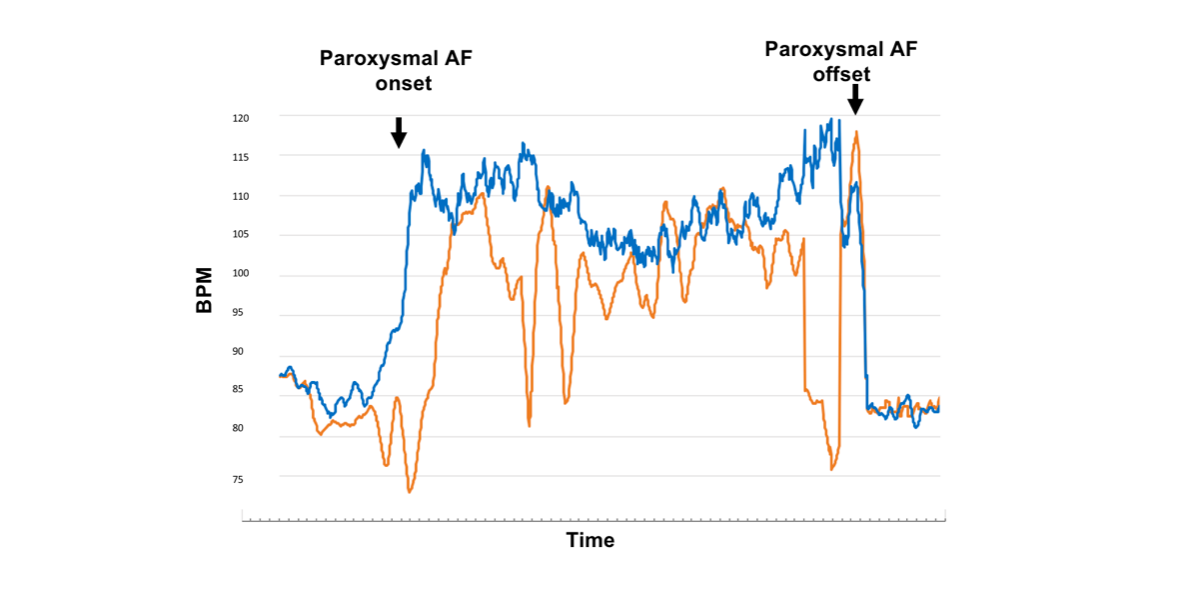
Time series trend curves during atrial fibrillation (event number 18). Figures 3 and 4 represent the same event (event number 18). Note that the 2 graphs differ in time intervals. This figure compares the trend curve of the Fitbit pulse rate (orange curve) with that of the electrocardiography-based heart rate (blue curve). Although the trends were statistically similar as a whole, brief episodes of an inverse correlation were present, thus weakening the correlation between the 2 curves (cross-correlation function 0.55; *P*&lt;.001). AF: atrial fibrillation; bpm: beats per minute.

Figures 3 and 4 represent an event (number 18) and show the 2 time series curves related to this event: one representing heart rate trend and the other representing pulse rate trend. The CCF analyses revealed that the trend patterns during this event were almost identical between AWW and ECG (Figure 3; CCF 0.83, *P<*.001) and were similar as a whole but having brief episodes of negative correlation (or inaccurate pulse rate measurements) between FBT and ECG (Figure 4; CCF 0.55, *P*<.001).

**Figure 5:**
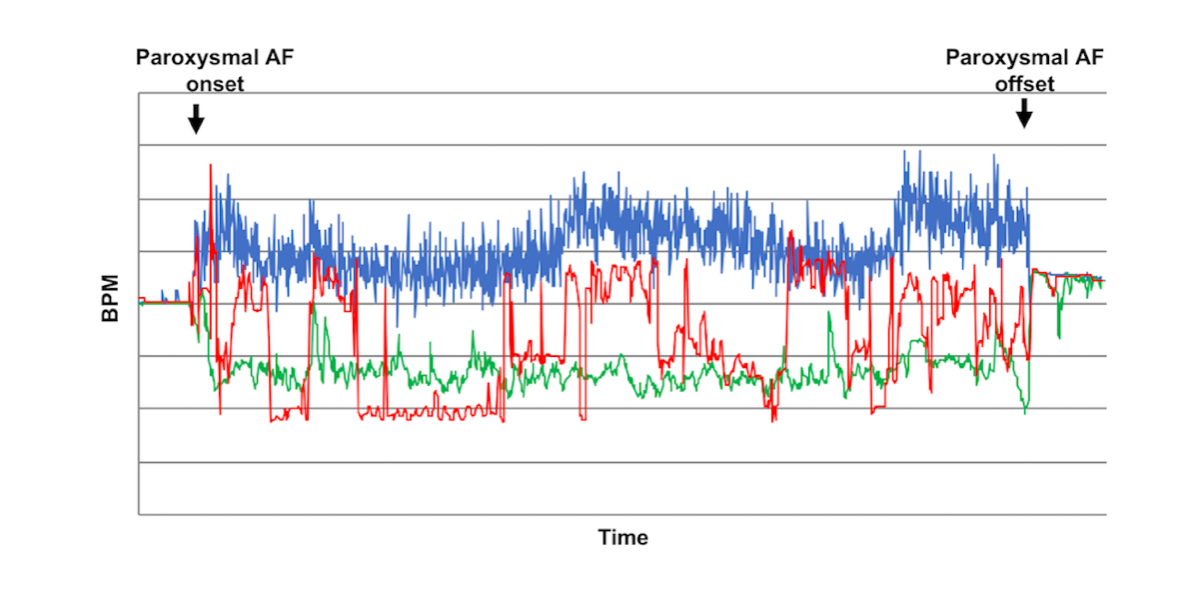
Time series trend curves during atrial fibrillation (event number 22). The 3 trend curves (electrocardiography heart rate, blue curve; Apple Watch W mode pulse rate, red curve; and Fitbit pulse rate, green curve) were compared for similarity. The subject patient who experienced this event was hypotensive at the time of the event. Both Apple Watch W mode and Fitbit showed a negative correlation for pulse rate trends when compared with the heart rate trend (cross-correlation function −0.02 and −0.62, respectively; *P*=.28 and &lt;.001, respectively). Note that the negative correlation was stronger and significant with Fitbit. AF: atrial fibrillation; bpm: beats per minute.

Figure 5 also represents an event (number 22) and shows the trend curves that resulted as a negative correlation for both AWW and FBT (CCF −0.02 and −0.62, respectively; *P*=.28 and <.001, respectively). Note that the negative correlation was stronger and significant with FBT. The subject patient who experienced this event was hypotensive (systolic pressure of 80-85 mmHg) at the time of the event and had low left ventricular ejection fraction (0.34) before surgery. Soon after this event, the same patient had another AF event (number 23), which similarly showed a very weak or negative correlation for both devices.

**Table 2:** Time series correlation of pulse change in paroxysmal atrial fibrillation.

Event number	Cross-correlation function			
	Apple Watch workout mode^a^	*P* value	Fitbit Charge HR^a^	*P* value
1	—^b^	—	0.13	<.001
2	—	—	0.54	<.001
3	—	—	−0.04	.04
4	—	—	0.20	<.001
5	—	—	0.49	<.001
6	—	—	0.25	<.001
7	—	—	0.02	.001
8	0.81	<.001	0.62	<.001
9	0.71	<.001	−0.08	<.001
10	0.68	<.001	0.41	<.001
11	0.79	<.001	0.37	<.001
12	0.45	<.001	—	—
13	0.23	<.001	—	—
14	0.36	<.001	0.13	<.001
15	0.59	<.001	0.02	.06
16	0.51	<.001	—	—
17	0.64	<.001	0.39	<.001
18	0.83	<.001	0.55	<.001
19	0.83	<001	0.71	<.001
20	0.78	<.001	0.38	<.001
21	0.85	<.001	−0.35	<.001
22	−0.02	.28	−0.62	<.001
23	0.02	.22	−0.38	<.001

^a^For reference, the strength of correlation [34] can be classified in the literature as: <0.19, very weak; 0.2 to 0.39, weak; 0.4 to 0.59, moderate; 0.6 to 0.79, strong; >0.8, very strong.

^b^Apple Watch workout mode was not used during the period when some events (numbers 1-7) occurred; hence the missing values for those events. Other missing values represent unavailable data.

### Validation of Accuracy of PPG-Based Pulse Rates During Atrial Fibrillation: Simple Linear Regression Analysis

**Figure 6:**
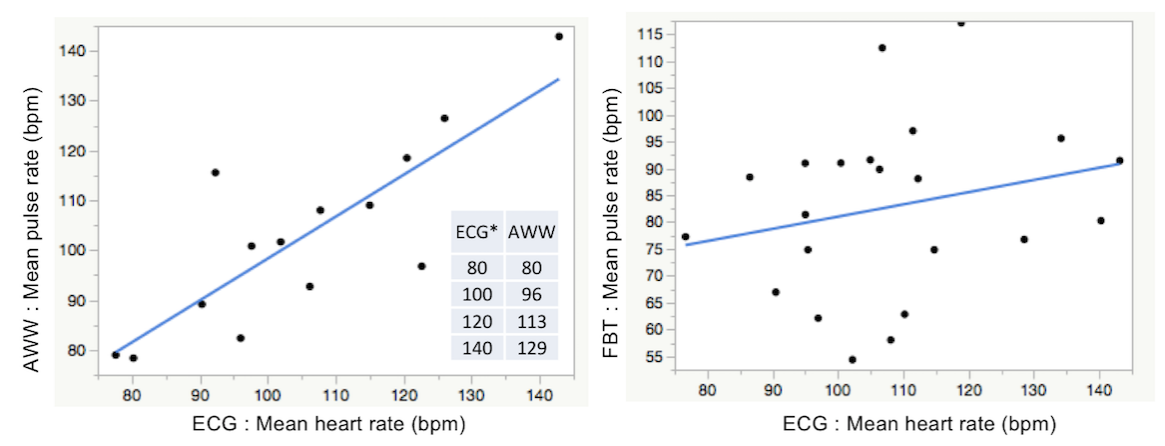
Scatter plot and simple linear regression analysis for the mean of all average pulse rates and all corresponding average heart rates during atrial fibrillation. AWW: Apple Watch workout mode; bpm: beat per minutes; ECG: electrocardiography; FBT: Fitbit Charge HR.

**Figure 7:**
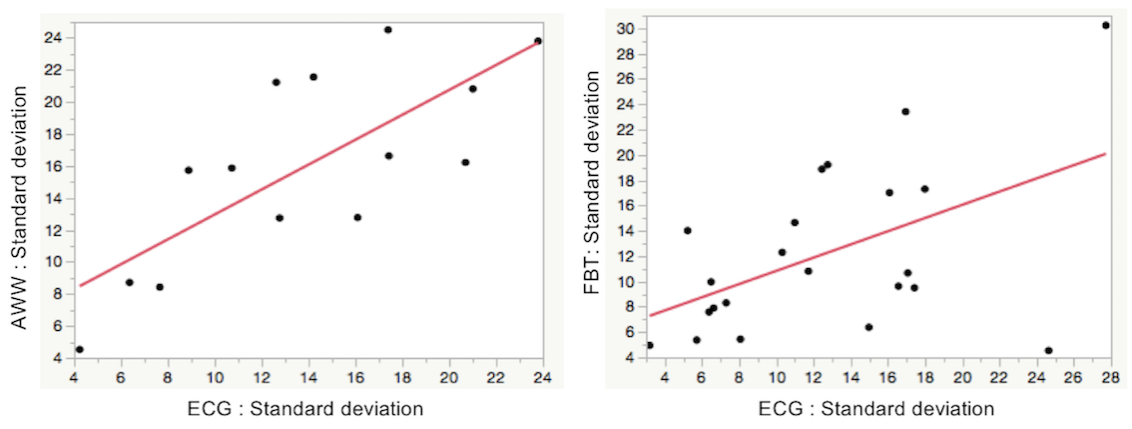
Scatter plot and simple linear regression analysis for the standard deviation of all average pulse rates and all corresponding average heart rates during atrial fibrillation. AWW: Apple Watch workout mode; bpm: beat per minutes; ECG: electrocardiography; FBT: Fitbit Charge HR.

The formulas for the fitted regression lines for both the mean and the standard deviation of all average pulse rates and all corresponding average heart rates were obtained using the linear regression model. The scatter plots and the regression lines derived from the regression analysis are shown in Figures 6 and 7.

Where *X* denotes the mean of all average pulse rates during AF and *Y* denotes the mean of all corresponding average heart rates during AF, the regression formula for AWW and FBT was *X*=14.203 + 0.841*Y* and *X*=58.225 + 0.228*Y*, respectively, and the coefficient of determination (*R*^2^) was 0.685 and 0.057, respectively (*P*<.001 and .29, respectively).

Where *A* denotes the standard deviation of all average pulse rates during AF and *B* denotes the standard deviation of all corresponding average heart rates during AF, the regression formula for AWW and FBT was *A*=5.178 + 0.778*B* and *A*=5.610 + 0.522*B*, respectively, and *R*^2^ was 0.572 and 0.255, respectively (*P*<.002 and .02, respectively).

From these analyses, the pulse rate data obtained from AWW significantly reflected the heart rate data from ECG, whereas this correlation was not found with FBT. However, an incremental increase in the difference between the pulse rate and the heart rate was observed as the rate increased in the AWW group.

## Discussion

### Principal Findings

Despite the rapid growth and improvement in PPG technology, there has been no direct comparison between long-term monitoring of pulse rates using a PPG device and that of heart rates by ECG in patients with paroxysmal AF. In view of this, we have started a project that evaluated the diagnostic feasibility of PPG-integrated smart watches for paroxysmal AF and to develop an algorithm for immediate detection and diagnosis of the arrhythmia using those smart watches as wearable monitoring terminals. The first part of the project was to conduct a validation study, a test of precision and accuracy of the PPG-based measurements during AF. This was done by using the time series data of the pulse rates and matching those with the corresponding time series data of the heart rates. Owing to the difference in the algorithm for pulse measurements between devices, we also compared the 2 most common devices currently available in the market.

The main findings of this validation study were as follows: (1) paroxysmal AF can be detected with sufficient precision from the trends of the pulse rates, although some adjustments may be required (eg, during unstable hypotensive states); (2) pulse rates based on PPG measurements can be matched with heart rates from ECG with sufficient accuracy, although some adjustments may be required (eg, during tachycardia); and (3) AWW has the highest precision and accuracy with regard to AF detection and pulse measurement during AF compared with FBT and the S mode of Apple Watch. It is important to note that these findings were accentuated by the following characteristics: (1) the study was performed under an environment where the subjects were allowed ambulation, (2) the comparison between PPG-based data and ECG-based data was done continuously over a long observation period, and (3) the onsets and offsets of AF were analyzed based on PPG data. These characteristics formed the backbone of this validation study and are crucial for the next step of this ongoing project.

In this validation study, a positive correlation was found between the trend patterns of the heart rates and the pulse rates during AF, implying that AF can be tracked with sufficient precision using a PPG device and that PPG device users can possibly be alerted at the onset of AF. However, there were incidents where an inverse correlation was found, suggesting a potential limitation to the reliability of the device if used under certain conditions. One such condition is low blood pressure. Reduced peripheral blood pressures may weaken the pulse signals that can be captured by PPG. Similarly, rapid AF is known to adversely affect cardiac output and cause malperfusion of the peripheral vasculature [35-37]; thus, further research may be required to test whether other pathophysiologic conditions, such as rapid AF, will compromise the diagnostic capability of PPG.

Regarding the accuracy of the pulse measurements during AF, the regression line formulated from the linear regression model showed that when AWW is used, a near linear relationship between pulse rates and hearts rates existed. However, there was an incremental increase in discrepancy between the estimated pulse rate and the heart rate as the rate increased, implying that there may be a limit for which pulse rate computed on the smart watch can accurately reflect the heart rate on ECG [38].

In this study, we also evaluated whether the dispersion of the pulse rates correlated with that of the heart rates during AF. This was done by comparing the standard deviations of both measurements. Unlike other arrhythmias with a fixed RR interval, AF exhibits standard deviations that are inconsistent and thus scattered when plotted on a time series scatter diagram. This inconsistency may help differentiate AF from other common arrhythmias in clinical practice. In terms of validation, we found a positive correlation in the standard deviations between AWW and ECG (Figure 7), thus adding to the suitability of this PPG device for further research.

### Comparison With Prior Work

Previous related studies have reported similar findings. In a study using a different model of Apple Watch and FBT, Koshy et al [26] demonstrated that a rate of more than 100 beats per minute (bpm) would result in a difference of 40% and 85% for Apple Watch and FBT, respectively, between heart rate and pulse rate. Similarly, other studies [39] have shown a discrepancy between electrical ventricular rate and pulse rate during AF in clinical practice, likely reflecting an occasional absence of aortic valve opening during rapid conduction of electrical impulse within the ventricular myocardium. In an animal model of AF, this discrepancy accounted for a 96.8% reduction of effective ventricular rate, or pulse rate, when the electrical ventricular rate was 80 bpm and a 92.5% reduction when the rate was 120 bpm [40].

### Limitations

This study has a number of limitations. The sample size was small, and the subjects of this study were elderly patients who required cardiac surgery. All of the subjects were on medications with some subjects in unstable hemodynamic conditions. Thus, the study was directed at people with limited movement and low activity and did not account for motion artifacts in daily life [41]. However, all of those issues will be addressed in our next study.

PPG is affected by multiple factors, including measurement location, skin conditions, and tightness of skin contact [42]. In this validation study, the differences in results between devices were unlikely to be because of those factors, as the devices were worn side by side and on the same side of the wrist. Apple Watch has 4 PPG sensors and an automatic luminance regulation system [31], and FBT has only 2 sensors and does not have the auto adjustment function, and thus, it is likely that device performance itself was responsible for the differences.

### Clinical Prospects

In recent years, industries have begun shifting their production toward wearable devices equipped with a portable electrocardiograph. The new Apple Watch Series 4 carries an electrical heart sensor that, when used with an app, generates a single-lead electrocardiogram capable of diagnosing AF [43]. The electrocardiogram is generated by bringing both hands (the wrist and a finger) in contact with the device; thus, the diagnosis of AF is possible only when AF is present. This feature is particularly useful when AF can be detected on the spot using PPG [44]. By combining those technologies with the rapidly evolving telemedical services and artificial intelligence technology and the emergence of direct oral anticoagulants that do not require routine blood monitoring, public health care may enter a new era encompassing efficiency and efficacy, particularly with regard to stroke prevention [43-48]

### Conclusions

This first part of the 2-phase study showed that PPG-integrated smart watches can reliably detect AF under controlled conditions. On the basis of this study, AWW was considered most suitable for the detection of paroxysmal AF. The device demonstrated optimal performance in both detection precision and measurement accuracy when AF occurred. Our next step is to use these data to achieve our purpose of this study—the development of an algorithm for on-the-spot detection and diagnosis of paroxysmal AF using artificial intelligence technology to facilitate and enhance the detection performance.

## Abbreviations

AF: atrial fibrillation
AWS: Apple Watch S mode
AWW: Apple Watch W mode
bpm: beats per minute
CCF: cross-correlation function
ECG: electrocardiography
FBT: Fitbit
PPG: photoplethysmography
S: standby
W: workout
